# Disquisitions Relating to Principles of Thermodynamic Equilibrium in Climate Modelling

**DOI:** 10.3390/e24040459

**Published:** 2022-03-26

**Authors:** Leslie V. Woodcock

**Affiliations:** Department of Physics, University of Algarve, 8005-139 Faro, Portugal; lvwoodcock@ualg.pt

**Keywords:** climate modelling, thermal equilibrium, atmospheric thermodynamics, adiabatic expansion, lapse rate, troposphere, radiation balance

## Abstract

We revisit the fundamental principles of thermodynamic equilibrium in relation to heat transfer processes within the Earth’s atmosphere. A knowledge of equilibrium states at ambient temperatures (T) and pressures (p) and deviations for these p-T states due to various transport ‘forces’ and flux events give rise to gradients (dT/dz) and (dp/dz) of height z throughout the atmosphere. Fluctuations about these troposphere averages determine weather and climates. Concentric and time-span average values <T> (z, Δt)) and its gradients known as the lapse rate = d < T(z) >/dz have hitherto been assumed in climate models to be determined by a closed, reversible, and adiabatic expansion process against the constant gravitational force of acceleration (g). Thermodynamics tells us nothing about the process mechanisms, but adiabatic-expansion hypothesis is deemed in climate computer models to be convection rather than conduction or radiation. This prevailing climate modelling hypothesis violates the 2nd law of thermodynamics. This idealized hypothetical process cannot be the causal explanation of the experimentally observed mean lapse rate (approx.−6.5 K/km) in the troposphere. Rather, the troposphere lapse rate is primarily determined by the radiation heat-transfer processes between black-body or IR emissivity and IR and sunlight absorption. When the effect of transducer gases (H_2_O and CO_2_) is added to the Earth’s emission radiation balance in a 1D-2level primitive model, a linear lapse rate is obtained. This rigorous result for a perturbing cooling effect of transducer (‘greenhouse’) gases on an otherwise sunlight-transducer gas-free troposphere has profound implications. One corollary is the conclusion that increasing the concentration of an existing weak transducer, i.e., CO_2_, could only have a net cooling effect, if any, on the concentric average <T> (z = 0) at sea level and lower troposphere (z < 1 km). A more plausible explanation of global warming is the enthalpy emission ’footprint’ of all fuels, including nuclear.

## 1. Introduction

The subject of classical thermodynamics provides a description of the properties of all pure elements and compounds, multicomponent mixtures, and colloidal fluids. The earth’s lower atmosphere, the troposphere, that determines climates is a multicomponent mixture of gases, and in part, a colloid of water in air. In an idealized system state known as the thermodynamic limit, the system is deemed to be infinitely large so that there are no surface effects or finite size effects, and all molecular fluctuation-correlation timescales are deemed to be so short that all such systems in this limit are truly homogeneous. Systems that are inhomogeneous in the thermodynamic limit either in space or time are generally described as being thermodynamically “small”. In this context, the atmosphere is a “small” system.

In the thermodynamic limit, before the first and second laws of thermodynamics can be formulated, there needs to be a formal definition of the two principles of mechanical equilibrium and thermal equilibrium proposed originally by Isaac Newton in 1687 and Joseph Black in 1850, respectively. Only then, can we define state functions of p-T that determine the first and second laws of thermodynamics, simply stated Q_rev_ (=ΔH enthalpy change) and Q_rev_/T (=ΔS entropy change), respectively [1]. Q_rev_ is reversible heat transferred to or from a system in a reversible process. Whilst real systems and real processes all involve deviations from these well-defined equilibrium states, a proper understanding of the underlying thermodynamic equilibria is fundamental to an understanding and description of real systems and irreversible processes, which, to some degree, are thermodynamically small in a gravitational field.

There are various reasons for thermodynamic “smallness”, colloidal particles with long time scales, multiphase systems in weak field, systems of low dimensionality, and many different types of thermodynamically small systems of interest in nature. When a gravitational field is applied to a thermodynamic system such as a molecular fluid, it is trivial to show that the gravitational force on each individual molecule is many orders of magnitude less than the root mean square fluctuating intermolecular force and is hence negligible.

As a result, even though the gravity mainly determines the pressure profile of the Earth’s atmosphere as a function of height and the pressure of the oceans as a function of depth, the thermodynamic properties of the atmosphere at a particular height or the ocean pressure as a function of depth are reasonably accurate from the known equilibrium equations of state. Thus, the effect of smallness arising from an external field in a condensing multicomponent system is predictable in the limits of high and low densities. However, system smallness can also arise because of finite extent of the system in the direction of a strong external field.

We can describe the Earth’s atmosphere as a thermodynamically small system because characteristic fluctuation lengths ~ km+ are of the same order as the characteristic length scales of the atmosphere under gravity. On the other hand, ironically, a one-liter sample of pure air, say, in both thermal and mechanical equilibrium with uniform T and p, a gradient in T or p of the atmospheric lapse rates 0.0001 K/cm may be negligible, i.e., sufficiently close to the thermodynamic limit and not thermodynamically “small”. In a highly inhomogeneous non-equilibrium system as complex as the Earth’s atmosphere, local processes with length scales of kilometers such as wind and rain are predictable. Clausius law [1] for irreversible processes tells us that for all spontaneous processes state functions change in the direction towards thermodynamic equilibrium of increasing entropy to a maximum. Thermodynamics, moreover, given the thermodynamic state functions ΔH and ΔS of all the components, can tell us what the overall direction of a spontaneous physical or chemical change is and whether a transformation occurs.

Given the state functions of its components, thermodynamic laws can tell us what the equilibrium state of the Earth’s atmosphere would be if the Earth stopped rotating and orbiting, the sun stopped shining, volcanoes stopped erupting, the oceans stopped evaporating, radioactive active material stopped decomposing, etc. One must be cautious in drawing conclusions from any predictive computer model when all these external and internal processes conspire to create a truly chaotic system. The local state variables, T and p, of space and time are literally fluctuating on all length scales from millimeters to kilometers to time scales from minutes to millions of years. Yet, we can look out the window and predict the weather for the next hour or so. Given modern technology, instantaneous knowledge of several thousand thermometers and barometers strategically placed around the Earth’s surface, and the most powerful computers imaginable, computer weather forecast modelling cannot extend this prediction with any certainty beyond a few days because the atmosphere is a chaotic, stochastic, and multivariate system.

The application of principles of thermodynamics to “small systems” began in the 1960s when Hill derived general equations applicable to non-macroscopic systems, which in the limiting case of an infinite system would reduce to “ordinary thermodynamics” [2,3]. Instead of deriving the fundamental relations between thermodynamic quantities in the various statistical mechanical ensembles for application to small systems, an alternative more useful and modern approach is to adopt a quasi-thermodynamic approximation and to use density functional theory (DFT) [4,5,6]. 

Here, we investigate hypotheses of thermodynamic equilibrium processes in the predictions of thermodynamic state averages of the Earth’s atmosphere which are commonly used in climate change science modelling [7,8] to estimate the effect on average temperatures of increased concentrations of (so-called) greenhouse gases that transduce light into heat. These open-system sub-states are thermodynamically small because their thermodynamic T-p state profiles are determined by externally imposed gradients of pressure by gravity and temperature by radiation energy transfer. 

## 2. Experimental Criteria

### 2.1. Global Average Temperatures

The atmosphere can be treated as a multi-component single Gibbs phase of N_2_, O_2_, CO_2_, Ar, and homogeneous mixture with variable water concentration (H_2_O) at states up to a small humidity that exists at pressures from 1 atmosphere at the Earth’s surface to vanishingly small beyond the thermosphere (100 km). The thermodynamic state variables are permanently in a state of flux at all points in the system. One can define averages of temperature (T) at any point in space as a function of latitude (φ), longitude (θ), height (z), and time (t), i.e., < T> (φ, θ, z, Δt). The angular brackets denote an average over a time span Δt that, without a seasonal specification, is not less than one year to include all fluctuations arising from the Earth’s daily rotation and annual orbit. “Global average temperatures”, a concept widely used in climate change theory, are variously defined operationally as an average of measurements over the Earth’s land and sea surfaces.

**Figure 1:**
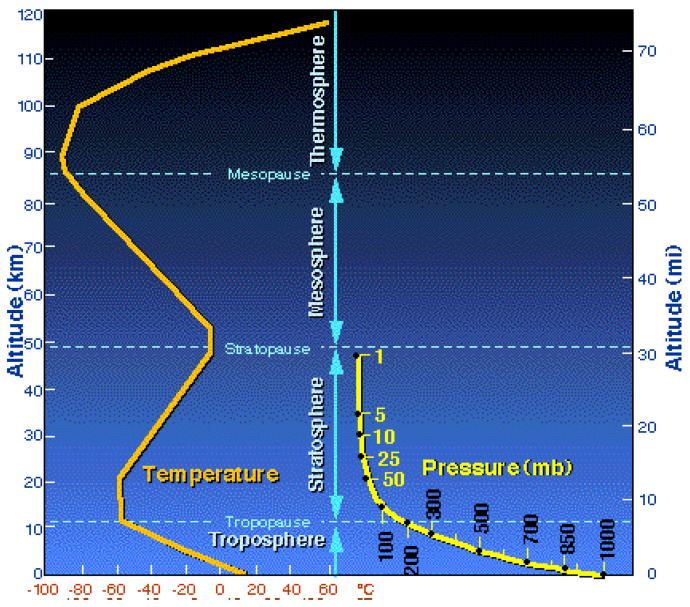
Experimental mean temperature (T) and pressure (p) profiles of the Earth’s atmosphere; the density functional profile < ρ(T, p)_z_ > is indicated by the increase in darkness with height; yellow lines for temperature (T) and pressure (p) are concentric averages denoted by angular brackets in the text. https://okfirst.mesonet.org/train/meteorology/VertStructure2.html (accessed on 20 December 2021).

A basic objective of any climate model based upon atmospheric thermodynamics is to predict the experimental profiles of the global concentric time averages of the temperature < T> (z, Δt) and <p > (z, Δt). Typical profiles of long-time averaged T(z) and p(z) from meteorological research are shown in Figure 1.

### 2.2. Barometric Pressures

Since the atmosphere is not contained, except for gravity, the pressure profile is predicted accurately using the ideal gas equation of state (pV = RT where R is the gas constant and T is absolute K). The maximum ground-zero mean sea-level air pressure of 1 atmosphere is exactly known, and the ideal gas equation of state p = RT/V holds at that and all lower pressures. Figure 1 also shows that the average temperature up to around 100 km is everywhere below the 300 K (27 °C) but on average only by around 10%. To a first approximation, T(K) is constant assumed T = <T> (z) where the local density approximation of DFT applies, and both the pressure and the density profiles are obtained from the ideal gas equation of state. The result for the pressure profile is generally called the *barometric formula*; it is in quite good agreement with the experimental logarithmic curve shown in Figure 1.

RT log_e_ (p/p_0_) = RT log_e_ (ρ/ρ_0_) = −gmz (1)

where m is the molar mass of air and ρ is a density.

Substituting for RT = pV and differentiating with z gives an approximate atmospheric density profile.

ρ(z)μ = −{(dp/dz)_T_}(z)/g (2)

We note here that the mean air density decreases approximately linearly with z in the troposphere as does the concentration (denoted by square brackets: mol/L) of its main components [N_2_], [O_2_], [H_2_O], [Ar] and [CO_2_] to a first approximation. A corollary of this observation is that the z-dependence of any absorption or emission of electromagnetic radiation, being proportional to concentration, also varies linearly with z.

### 2.3. Experimental Observations

The experimentally measured decrease in <T>(z) of the troposphere is approximately constant around −6.5 K/km. This quantity plays a central role in climate modelling [7,8] but its scientific origin appears to be the subject of a misrepresentation of the principles of equilibrium thermodynamics. The theory giving rise to its calculation in the troposphere appears to be based upon the misconception, that its existence is determined by a thermodynamic reversible adiabatic expansion or compression of volume elements of the Earth’s atmosphere. There can be no thermodynamically reversible adiabatic expansions or contractions occurring in the atmosphere since it coexists with the Earth’s surfaces with no adiabatic or closed boundaries.

The experimental evidence suggests the atmospheric lapse rates in the atmosphere are not a consequence of thermal equilibrium. A thermal equilibrium state of a system under gravity must have a uniform temperature with the lapse rate d <T>/dz = zero. The observed oscillations of the sign of the mean temperature gradient in Figure 1 must be determined primarily from non-equilibrium heat balance radiation absorptions and emissions in the first instance. The concept of a “parcel of air” in the atmosphere undergoing a reversible adiabatic expansion to create the temperature gradient, which can only occur in a closed system, may account for fluctuations around <T> (z) and affect weather events that we consider later. First, we reviewed the prevailing assumption [9] that the lapse rate is determined by adiabatic expansion of an otherwise equilibrium isothermal fluid state of air, say, a surface temperature T_0_.

### 2.4. Lithosphere Temperature Profile

**Figure 2:**
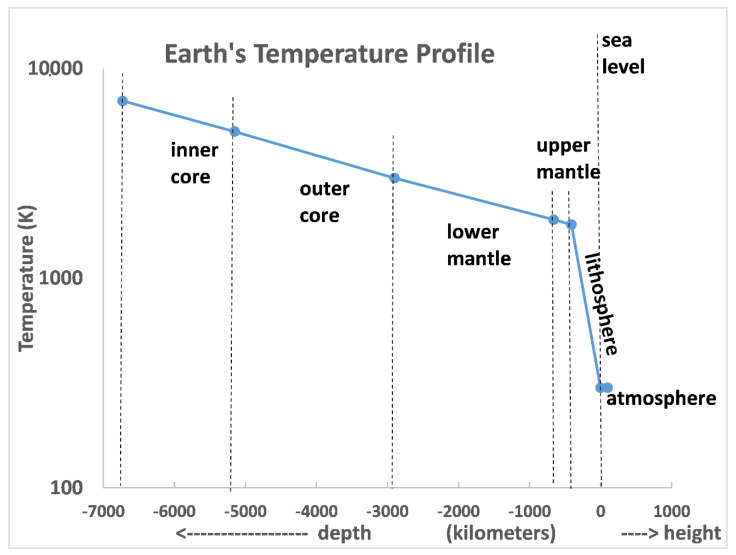
Mean temperature profile of the Earth and its atmosphere; the average temperature of the atmosphere is around 300 K: the average gradient <dT/dz> for the lithosphere is around –5 K/km, i.e., slightly less than the troposphere average up to the tropopause at 10 km (Figure 1): the atmosphere outer limit is 100 km.

To explain the experimental troposphere lapse rate up to 10 km, consider the global lapse rate of the whole Earth. Its crust, the lithosphere with a depth of ~ 400 km, and atmosphere with a height of 100 km (Figure 2) shows that the Earth itself is cooling from its core by around 1 K/km but that the cooling rate suddenly increases to around –5 k/km on average for the Earth’s lithosphere to a depth of around 400 km. At sea level (T_0_), the Earth’s solid crust lapse rate is closer to the experimental atmospheric lapse rate of –6.5 K/km than the DARL value –9.8 K/km. If we extend the outer limit of the average lithosphere lapse rate to the global mountainous extremities at z ~ 9 km, we see from Figure 2 that the linear constant troposphere lapse rate seen in Figure 1 up to the tropopause at 10 km can explain to a degree the experimental average lapse rate. The ground temperature at z ~ 9 km is roughly 50 K below the mean sea-level global average ground temperature, as also is the ambient atmosphere at that level. Heat transfer by transverse convection (winds) have a moderating effect to some extent upon the mean lapse rate < dT/dz > (z) in the troposphere.

Whereas the solid Earth’s lapse rate is monotonic everywhere, the atmospheric lapse rate is oscillatory in sign and the changeovers are used to classify the concentric regions of different thermodynamic behavior. <dT/dz> changes from negative to positive from troposphere to stratospheres and back to negative again for the mesosphere, then back to positive for the thermosphere. In the thermosphere around 100 km and higher, the temperature appears to be ever-increasing as it approaches p ~ 0 in outer space. This begs the question: what causes the reversals in sign of (d <T>/dz)z on climbing from troposphere to stratosphere, stratosphere to mesosphere, and mesosphere to thermosphere? 

## 3. Lapse Rate in Climate Modelling

### 3.1. Adiabatic Lapse Rate

**Figure 3:**
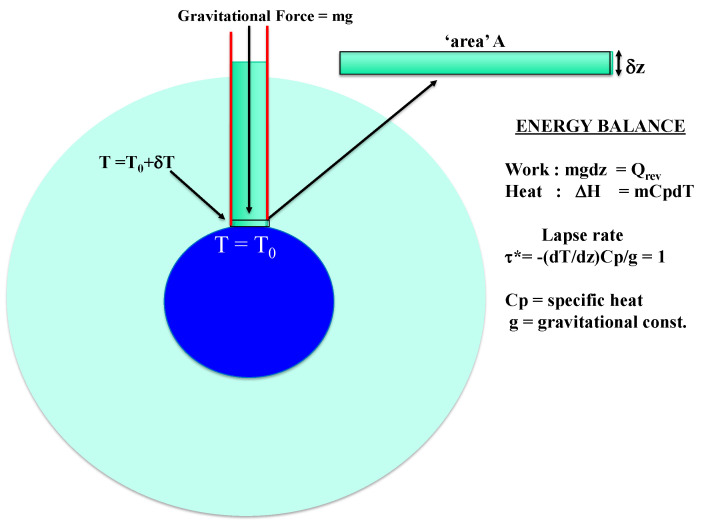
Schematic 2D illustration of an energy balance for a hypothetical reversible adiabatic expansion process for an infinitesimal expansion of a column volume element of the Earth’s atmosphere, initially at equilibrium at temperature T, by an amount Adz (=dV) at the local gravitational pressure p(z): the hypothetical expansion in isolated closed system indicated by red lines in equilibrium with T_0_ at ground zero with no roof under gravity would predict a non-equilibrium constant gradient in temperature (dT/dZ) known in climate modelling as the “dry adiabatic lapse rate” (DALR) = –9.8 K/km [7,8,9].

To calculate a dry adiabatic lapse rate (DARL), defined using the heat capacity of dry air, climate modelers hypothesize [7,8,9] that for a “parcel of air, within a still vertical column at equilibrium” with just the gravitational hydrostatic pressure, zero at the top, and height z → infinity, the pressure at the base z = 0 is equal to gm/A with the thermodynamic limit in x,y planes being open and in thermodynamic limit. However, if this hypothetical model atmosphere existed (see Figure 3) at the ground temperature T_0_ at thermodynamic equilibrium, the temperature of the column would be uniform with T(z) = T_0_ everywhere and the lapse rate (dT/dz) would equal zero for all values of z. 

The lapse rate adiabatic hypothesis considers this representative sample column of gas to change states in uniform adiabatic expansion under the force of gravity such that the volume occupied by an infinitesimal ‘pizza’ slice of height z and width dz increases by an amount dV. The work done by this adiabatic expansion is simply pdV, where p is the pressure at level z. The pressure at level z is the force of gravity x weight (for an ideal gas, one can use the barometric formula), and since the process obeys Joules law [1] for all processes, reversible and irreversible), work performed against surroundings = enthalpy change; for a simple energy balance for an infinitesimal change dp, we have for the enthalpy of work performed (by definition and/or Joule’s law [1]):

H = −Q_rev_ = W_rev_ = mC_p_dT (3)

where C_p_ is the specific heat per unit mass (m), W is work, and Q is heat; work to change pressure is:

W_rev_ = Vdp (4)

then, using barometric Equation (2) dp = gρdz where density ρ = m/V, adiabatic lapse rate is:

(dT/dz) = g/C_p_ (5)

The result can equally be obtained as shown schematically in Figure 3: since for an ideal gas, pdV = −Vdp for any infinitesimal expansion. This simple energy balance produces a non-equilibrium temperature gradient for a mechanical equilibrium of a uniaxial expansion of a subvolume Adz of air that is isolated and closed to the surrounding atmosphere. The result is that (dT/dz)_z_ for any isolated gas expanding reversibly and adiabatically under gravity, if the process were possible, would be a material constant that depends only upon its heat capacity C_p_.

### 3.2. Troposphere Lapse Rate Hypothesis

There is a well-documented belief, especially amongst the climate modelling community [7,8], that because of this result, g/C_p_ for dry air = –9.8 K/km appears to agree with the experimental observation of a linear negative lapse rate in the troposphere of the same order. Figure 1 shows that <T> (z) varies from 15 °C (288 K) at z = 0 to –50 °C (223 K) at z = 10 km, giving an experimental lapse rate < (dT/dz) > (z) = –6.5 K/km, i.e., somewhat less than the adiabatic hypothesis constant. Because of this rather coincidental result, it has been generally assumed that an average temperature <T> (dz), say, the global concentric average near sea-level, is a result of natural convection from a radiation-heated Earth’s surface (~70% oceans and 30% land). Sunlight hits the surface of the Earth (land and sea) and heats them. They then heat the air above the surface. This explanation for the negative troposphere temperature gradient is summarized: (from Wikipedia [9], see also references [10,11,12] for further detailed analysis).

*QUOTE:* “However, when air is hot, it tends to expand, which lowers its density. Thus, hot air tends to rise and carry internal energy upward. This is the process of convection. Vertical convective motion stops when a parcel of air at a given altitude has the same density as the other air at the same elevation. When a parcel of air expands, it pushes on the air around it, doing thermodynamic work. An expansion or contraction of an air parcel without inward or outward heat transfer is an adiabatic process. Air has low thermal conductivity, and the bodies of air involved are very large, so transfer of heat by conduction is negligibly small. Also, in such expansion and contraction, intra-atmospheric radiative heat transfer is relatively slow and so negligible. Since the upward-moving and expanding parcel does work but gains no heat, it loses internal energy so that its temperature decreases. The adiabatic process for air has a characteristic temperature-pressure curve, so the process determines the lapse rate. When the air contains little water, this lapse rate is known as the dry adiabatic lapse rate: the rate of temperature decrease is 9.8 °C/km The reverse occurs for a sinking parcel of air” [11,12].

### 3.3. The Experimental Evidence

Below, we list some empirical objections inter alia why the foregoing prevailing hypothetical explanation for the troposphere lapse rate is inconsistent with the principles of thermodynamic equilibrium. The fundamental objection, however, is that gravity cannot determine a temperature gradient via adiabatic expansion or contraction processes without violating the second law of thermodynamics. A formal proof is given in Appendix A.

1. The first obvious indication that the climate modelers [7,8,9] adiabatic hypothesis cannot be a causal reason for <T> z in the troposphere is that the average temperature <T> (z = 9 km) above Mount Everest or the Himalayas area for example, is approximately the same as the collateral air temperatures 9 km above the sea level [13]. Where does the upward moving parcel of hot, isolated, insulated, and expanding air come from? The ground temperature at the extremity of the lithosphere (Figure 2) is almost the same as the mean air temperature above the oceans (–50 °C at 9 km).

2. A thermodynamically reversible uniaxial adiabatic expansion of air cannot occur in an open system such as the atmosphere. The mean air pressure at any level z, whatever the ground level is below, is primarily determined by the weight above; the temperature variation is caused by radiation balance effects determined in part by transducer gas concentrations and hence the pressures. Mean temperatures <T> (z) are maintained by prevailing transverse convection fluxes, e.g., between land and sea and/or night and day, i.e., winds, and not uniaxial upward convection.

3. The adiabatic expansion hypothesis cannot explain why the lapse rate is negative in the troposphere then changes to positive values in the stratosphere and negative again in the mesosphere [14]. If the lapse rate were to be a phenomenon of equilibrium thermodynamics of the atmosphere associated with reversible adiabatic expansions, the effect would be present throughout. All adiabatic expansions of ideal gases are associated with a cooling effect; the lower the density, the closer to the ideal gas behavior. Figure 1 shows that the lapse rate in the stratosphere is a positive heating effect, yet p(z) decreases with height (z) in accordance with the barometric formula and in contradiction to any adiabatic contraction hypothesis.

4. No thermodynamic fluid in a uniform external gravitational field can be in a state of thermal equilibrium if there exists within it any gradients of temperature. Such a scenario violates the second law (Appendix A). This is Black’s principle of thermodynamic equilibrium [1] (referred to also as the ‘zeroth law’). It is inviolable and determines the direction of heat flow in all non-equilibrium spontaneous processes. Newton’s principle of mechanical equilibrium of no unbalanced forces [1] is equally inviolable, so if the (dT/dz) in the troposphere (Figure 1) is a steady state non-equilibrium phenomenon, there would also be non-equilibrium gradients in pressure, hence the existence of transverse heat and mass transfer by convection everywhere.

5. Around 70% of the Earth’s surface is water with a surface temperature that is near to contact air temperature T_0_ for the most part. The suggestions [8,9,10] that the altitude cooling rate is determined by a thermodynamic equilibrium phenomenon of a reversible adiabatic expansion of a contained and closed column of air against gravity lacks a driving force on most of the Earth’s surface for most of the time. 

6. The statement in the above Wikipedia quote from the climate modelling literature [7,8,9] “intra-atmospheric radiative heat transfer is relatively slow” cannot be further from the truth. All heat transfer by radiation occurs effectively and instantaneously at the speed of light. When the sun sets on a clear day with a blue sky, there is simultaneous change in the ground, sea, and air temperatures of the biosphere. The various radiation effects combine to create, in effect, an instantaneous z-dependent average thermostat that maintains T-gradients. The local density approximation of DFT, given the local state variables p and T, determine the dependent variable density ρ(z) using the ideal gas equation of state = p(z)/RT(z). Hence, there can be coincidental similarity with a sequence of temperature drops with a hypothetical column of reversible adiabatic expansion processes.

### 3.4. Radiation Balance Atmospheric Thermostat

The experimental slope of <T> (z) in the troposphere around –6.5 K/km in Figure 1 has no direct causal relationship with the DALR constant (–9.8 K/km) for the above reversible thermodynamic expansion of a closed gas system of dry air. Accordingly, the steady state heat balances can only be explained by the plethora of radiation, absorption and emission, and heating and cooling effects from both the Earth surface, the atmosphere itself, and sun to maintain the non-equilibrium steady state thermostat T(z). This must then include all the effects of transducer gases leading to the variable mean experimental average temperature gradients shown in Figure 1 of oscillating signs that define the concentric regions. 

**Figure 4:**
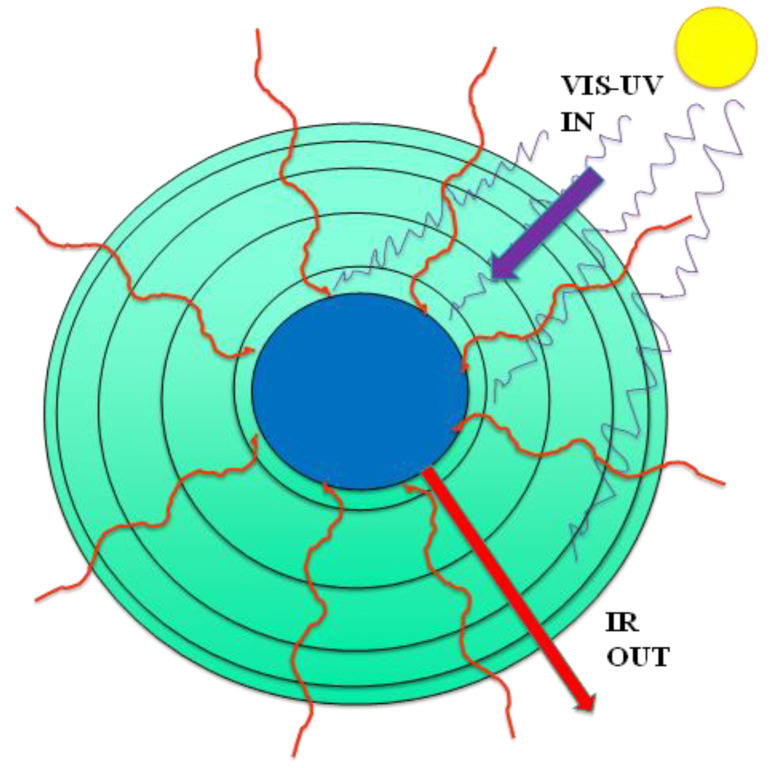
Schematic of a magnified 2D illustration of the atmospheric radiation balance ‘thermostat’ that determines the variant concentric average temperature as a function of height <T> (z) and its gradient <dT/dz> (z) of the Earth’s atmosphere: the heat transfer balance of the radiation is essentially instantaneous relative to the convective response relaxation processes, resulting in the near-constant negative lapse rate (d <T>/dz) in the troposphere (z = 0 to 10 km).

A temperature at any time of any volume element of the atmosphere T(z, θ, φ) is determined essentially instantaneously by the radiation balance at that radius (z) (Figure 4). We can regard the outcome of the radiation balance as a z-dependent “thermostat” that is effective on a time scale which is much shorter that the characteristic relaxation time for all other conductive and convective heat transfer processes. Accordingly, the LDA of density functional theory (DFT) [2,3] then applies to a volume element at the local equilibrium where the pressure, on average, is given by the barometric formula Equation (1) recalculated at a local <T> (z) and the density ρ(z) is given by Equation (2) as a function of T. 

This simplified scenario can explain the similarity between the mean lapse rate determined by the radiation balance and the subsequent adjustment of the atmospheric steady state temperature that is same sign and of same order as the DALR constant. In the troposphere, the intrinsic decrease in density is roughly the same as one expects for an adiabatic expansion since the local density approximation of density functional theory (DFT) [2,3] predicts a steady state change of T(z) with ρ(z) for an expansion process from air at T to T−dT and height z to z + dz. It has been suggested that the reason why the experimental mean lapse rate in the troposphere is 40% less than the DALR constant is the presence of water and its associated local heat and mass transfer effects such as enthalpy of condensation in cloudy skies and precipitation [15,16,17]. This has resulted in a revised and more general definition of a moist adiabatic lapse rate (MARL) that takes account of the change in properties of humid air. Water in the troposphere does indeed have a moderating and cooling effect due to its transducer properties such as the absorption and scattering of sunlight; these effects, however, have nothing to do with reversible adiabatic expansions.

The DALR dimensionless scaling variable (dT/dz)g/C_p_, which we denote by τ*, can be regarded as a local or transient consequence of fluctuations around the experimental lapse rate in Figure 1 but not the cause of it. Evidently, its value [7,8,9] is a stability limit that plays a similar role in atmospheric heat transfer by convective flow as do other transport stability limits, for example, Reynolds number in fluid flow in prediction the transition to turbulence.

## 4. Lapse Rate Instability Indicator

In DFT for small systems under gravity [2,3,4,5,6], temperature (T) is the equilibrium-independent state variable that determines the density (ρ) instead of the other way around. The adiabatic expansion hypothesis for the lapse rate in troposphere is based upon this misconception. This raises the question, is the adiabatic lapse rate constant and a material and physical constant scaling law that depends only on the heat capacity with any relevance in computer modelling or the prediction of events? The answer appears to be yes—it is a natural criterion for a gradient stability limit to be predicting the transition from laminar to turbulence for heat and momentum transfer instabilities in weather forecasting. However, it can be highly misleading when one asks the question of which factors, especially CO_2_, are influential in determining long-term variations in <T> (z).

There is abundant experimental evidence that the DALR constant 9.8 K/km is an instability limit wherever and whenever τ* exceeds a critical value. A large fluctuation from the mean is accompanied by fluctuations in density leading to an accompanying huge gradient in chemical potential unstable flow and mass transfer processes, leading to turbulence. It appears from an extensive catalogue of anecdotal evidence that this critical value is close to the DALR constant τ* = 1. Is this a coincidence? The evidence for the usefulness of τ* and its various modifications is in moist atmospheres that reduce its value by up to 50% at saturation [9]. This knowledge applied to modelling unstable weather phenomena such as turbulence is summarized below but there is no evidence that it is relevant to very small variations in <T> (z) with concentrations of greenhouse gases, e.g., CO_2_ (z) or H_2_O (z) in the troposphere.

Further compelling evidence that the lapse rate is a consequence of radiation thermostat and not uniaxial adiabatic convection processes is the longstanding observation that turbulence in the atmosphere is only found to occur in the troposphere ~0–10 km and not in the stratosphere ~10–50 km. Wherever the experimental lapse rate is lower than the τ* prediction, i.e., for all z higher than the tropopause height which is rather sharply defined by the change in lapse rate, atmosphere shows no turbulence. By contrast, the change from −7 K/km in the troposphere and stratosphere with a positive small temperature gradient (Figure 1) shows no turbulence [14]. The presence of water within the troposphere requires modification to the experimental interpretation of unstable events. If air rises due to the thermal-gradient-induced convection processes, it cools even more due to the heat of condensation. Eventually, clouds form, releasing the heat of condensation that further changes the local lapse rate as the clouds are powerful transducers of sunlight to heat. Before saturation, the rising air follows the dry adiabatic lapse rate. After saturation, the rising air follows the moist adiabatic lapse rate [15]. 

The exchanges of enthalpy and radiation energy balance fluctuations on cloud formation explains the occurrence of thunderstorms. Modified moist adiabatic lapse rates can be calculated with a lower value around 5 K/km [16,17]. Thus, for moist or saturated air in the troposphere, the threshold for instability where there is cloud formation is reduced by around 50% and the actual local lapse exceeds the moist adiabatic lapse rate (MALR), giving rise to turbulent weather patterns of wind and rain. Here, however, we were concerned with questions about changes in average concentrations of greenhouse gases, notably CO_2_ and H_2_O, on the concentric averages <T> (z) and d < T) >/dz (z). An analysis of time-dependent phase relationships between [CO_2_] (t) concentration and <T> (t, z = 0) showed a correlation over many decades with CO_2_ following <T> in the phase shift [18] rather than the reverse, as suggested by IPCC climate change models [7,8].

## 5. Climate Modelling

### 5.1. Computer Models of Atmospheric Processes

In the “dark ages” of science before the advent of computer modelling in the 1950s, scientific research was just theory and experiment. There are two types of approximation in theories, physical and mathematical. The advent of computer simulation enabled the numerical solution of multidimensional integrals, or high-order multi-coupled differential equations of otherwise intractable physical models. Building a physical computer model with superimposed mathematical approximations and hundreds of variable parameters is not scientific research. Computer modelling may have educational applications and engineering design uses or transient predictive applications such as weather forecasting, but such models cannot be a tool of scientific research. Computer “animation” of complex stochastic systems with hundreds of adjustable input parameters tells us nothing. 

In modelling the thermophysics of real systems, whatever the level—astronomical, phenomenological, molecular, electronic, or nuclear—in order to obtain new scientific information to test a hypothesis, we must perform what is known as Hamiltonian surgery. Hamiltonian is the mathematician whose name is given to the model definition of the energetics of the system. As in the laboratory, one can do an experiment on a computer model, but it is not what we put into a computer experiment that enables access to indisputable new knowledge, it is what we leave out. The simplest imaginable model of the thermodynamics of the Earth’s atmosphere is a gas, surface, and gravitational field that predicts a uniform temperature. This fails because it cannot predict thermal gradients as observed (Figure 1); the surgery is too drastic. Yet, the model has told us something.

The experimental lapse rate is evidence that conduction and convection, especially transverse convection, whilst important in moderating the natural fluctuations of T and p and all spontaneous weather processes, may not be significant factors in determining the experimental non-equilibrium concentric average < T(z) > lapse rate. If there were to be no radiation absorption or emission anywhere of any kind, then there would be no variations in ground temperature from poles to equator, from day to night, or summer to winter, and the entire atmosphere would come to thermal equilibrium at a mean temperature of the Earth’s surface <T> (z = 0) under gravity. Then, the simple barometric formula that assumes a constant T throughout would be precise for the pressure. Therefore, the next research step must include the simplest imaginable one-dimensional (z) homogeneous in x,y, model atmosphere with only the salient basic radiation transfer effects. 

### 5.2. Effects of H_2_O and CO_2_ on <T> (z)

**Figure 5:**
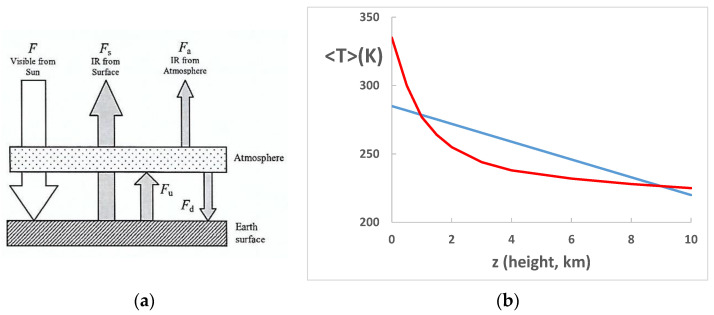
(**a**) Energy exchange in a primitive surface plus atmosphere one-dimensional two-level radiation balance model. (**b**) profiles of the model temperature from radiation balance in an atmosphere with no transducer gases (red line) compared with the real-world experimental linear lapse rate <T> (z) (blue line): note that <T> z = 0, i.e., at sea level is reduced from 335 K (62 °C) to 285 K (12 °C), and in the troposphere up to around 1.5 km by H_2_O absorption and, to a much lesser extent, CO_2_.

The most primitive model only considers Planck radiation exchanges between Earth’s surface, atmosphere, and sun in the form of radiation balance as summarized in Figure 5a showing the energy fluxes to and from an atmospheric volume element. The radiative flux F comes from the sun’s surface as visible or UV radiation The radiative flux F_s_ is emitted from the Earth’s surface and the atmosphere is also a black-body emitter which we call F_a_. We denote as F_u_ (upward flux) and F_d_ (downward flux) to describe the energy exchange between the Earth’s surface and the atmosphere occurring by all radiation processes. A more detailed description of a one-dimensional radiation balance model with a specification of parameters is given on page 211 of McGuffie and Henderson-Sellers, Section 4.2.1 [8].

Simply stated, the total emission from earth and atmosphere is (Figure 5a):

F = F_s_ + F_a_ (6)

The energy received from the sun according to the steady state radiation balance and lost by the Earth is constant total energy, as the long-term global temperature fluctuations are less than a fraction of 1K over hundreds of years. The Stefan–Boltzmann law determines the energies in radiation energy scales of Equation (6) as the fourth power of temperature [8,19]. This has a stabilizing effect on the exchange, and the flux emitted by Earth tends to be equal to the flux absorbed, close to the steady state. This result greatly simplifies the research question: what is the effect of changing the concentration of one or more of the atmospheric components on the concentric thermal average temperature <T> (z)? 

The likely effect, if any, on the steady state difference <ΔT(z)> due to a presence of CO_2_, for example, with all other things being equal, can be analytically obtained from the primitive model in Figure 5a. CO_2_ both absorbs and emits UV-visible radiation, so the additional CO_2_ warm up T(z) with the absorbed radiation, making the lapse rate smaller and hence <T> higher, maintained by radiation flux F_u_ and F_d_ balance. However, both F_s_ and F_a_ become larger than before according to the Stefan–Boltzmann radiation law [8,19] due to higher surface and atmospheric temperatures upsetting the steady state Equation (6). Then, both the surface and lower atmosphere cool down until they reach a new state of radiation balance, with Equation (6) once more satisfied. Likewise, UV or visible radiation from the sun is diminished by depletion of intensity at the absorption frequencies on passing through the atmosphere. This transducer (or greenhouse) gas absorption mainly by [H_2_O], but also [CO_2_], creates a heating effect at higher levels but a cooling effect of the lower troposphere and at the Earth’s surface (Figure 5).

If [CO_2_] increases, then it both absorbs and emits more radiation energy by amounts given by the Einstein coefficients [20]. At thermodynamic equilibrium, we have a simple balancing in which the net change in the number of any excited atoms is zero, being balanced by loss and gain due to all processes. This local thermodynamic equilibrium isothermal condition [20] requires that the net exchange of energy between different energy level states of the same component at the same temperature is zero. This is because the probabilities of transition cannot be affected by the presence or absence of other excited atoms, other components present, or the thermodynamic state. With the more emissive atmosphere at the higher T, the same total flux F_s_ + F_a_ occurs at lower temperatures; therefore, the concentric averages of <T> (z) can only be the same or lower with increasing CO_2_. This simple observation is not dependent on the absorption spectrum of CO_2_ but the magnitude of the effect is. Stronger atmospheric absorption of CO_2_ results in enhanced emission of the same component with everything else being equal and leads to a reduction of T(z) with decreasing z and of <T> (z = 0). The difference between the primitive model T profile, and the real-world lapse rate in the troposphere represents the total transduction effect of greenhouse gases, which is mainly due to water and CO_2_ at the lower levels of the troposphere.

We also note that the extensive specific heat C_p_ of air and CO_2_ that enters the estimates of DALR and MALR cannot affect the lapse rate effects, if any, due to adiabatic expansion. The change ΔC_p_ of one mole of air (all components) is due to an additional 0.0002 moles of CO_2_ (increase from 0.02 to 0.04%).

ΔC_p_ = Δ[CO_2_] {C_p_ (CO_2_)-C_p_ (air)} = 0.00005 C_p_(air) (7)

A higher C_p_ of air would correspond to a lower adiabatic lapse rate and an increase from T_0_ by 0.000005% according to Equation (5). This is clearly also a completely negligible effect. 

### 5.3. CO_2_ -Steam and Enthalpy ‘Footprints’

When a fossil fuel is burned, a hydrocarbon such as gasoline (octane) is replaced by 16 moles of CO_2_ and 18 moles of steam into the atmosphere roughly to every 25 moles of oxygen burned up. What would be the effect of this “CO_2_-H_2_O” footprint on <T> (z) if the exhausts were uniformly distributed in the troposphere? The balanced combustion chemical equation for octane is, showing a large enthalpy ’footprint’ for every 0.114 kg octane.

C_8_H_18_ + (25/2) O_2_ = 8 CO_2_ + 9 H_2_O − 5509 kJ (=ΔH) (8)

If we consider two identical-twin Earths, I and II: Earth I = no gasoline engines. Earth II = with gasoline engines, converting massive moles of fuel to [CO_2_ + steam]. What is the effect of gasoline engines on Earth II compared to Earth I, all other physical aspects of the solar system, atmosphere, biosphere, etc. being equal? 

It can be argued that the steam footprint is negligible compared to the water already present in the atmosphere at steady state and therefore its equilibrium with the clouds, oceans, biosphere photosynthesis, etc. all remain undisturbed by gasoline emissions over several decades. Adding more water vapor to the atmosphere, albeit relatively small, can produce a ‘steam footprint’ global cooling effect. This would happen because more water vapor leads to more extensive saturation in the troposphere, hence more cloud formation. Clouds reflect sunlight and reduce the amount of radiation heat that reaches the Earth’s surface to warm it. If the amount of solar warming decreases, then the temperature of the Earth’s surface and lower troposphere would decrease. In that case, the effect on average temperatures, if any, of adding more water vapor via the industrial ‘steam footprint’ that invariably accompanies the industrial ‘carbon footprint’ would more likely be global cooling rather than warming, but is probably a negligible effect.

Similar considerations, however, apply to [CO_2_]. A corollary of such an argument, therefore, is that if the steam footprint is negligible, then the CO_2_ footprint from gasoline engines is also negligible. Moreover, for [CO_2_], there is a balancing effect in the biosphere. Le Chatellier’s principle of equilibrium chemical thermodynamics [21] works toward restoration of the chemical equilibria for the photosynthesis reaction that removes CO_2_ from the atmosphere for plant growth, replacing the oxygen. If the concentration of [CO_2_] increases, everything grows faster. If the temperature of the biosphere (land surface) increases, everything grows faster. Both these effects, together with the CO_2_ output from humans and animals, eventually maintain a [CO_2_] balance.

A detailed analysis of the IR microwave absorption spectrum of CO_2_ by Witteman [22] was reported recently in relation to atmospheric absorption of Earth’s emission. The main conclusion was that since only 10% of its spectrum is active, the thermal radiation that falls within the frequency region of the Earth’s emission is fully absorbed. This is because there are only three fairly narrow absorption bands in CO_2_. This is not only the case for 400 ppm of CO_2_ in the atmosphere but also for much smaller concentration values. If computer model conclusions for global warming are dependent to any appreciable extent on IR thermal emission from the Earth’s surface, the heating effect is practically not affected by the change in the CO_2_ concentration since it is wholly absorbed at much lower concentrations in just the lower atmosphere layers. 

## 6. Conclusions

### 6.1. Thermodynamic Equilibria Summary

We found that *Black’s principle of thermal equilibrium*, alternatively known as the zeroth law of thermodynamics, plays a central role in determining the average temperatures and gradients in the various height spheres. It does not, however, determine the experimental lapse rates <dT(z)/dz> that are deviations from thermodynamic equilibrium due to the radiation balance which acts at a z-dependent thermostat, causing deviations from any global average temperature <T_G_> that would prevail if there was no radiation balance effect to create a non-zero experimental reduced lapse rate t * throughout the atmosphere. The τ * = 1 results for an idealized uniaxial adiabatic expansion against gravity is given by a simple energy balance between the gravitational force mg/A as there is no intrinsic pressure, and the enthalpy loss is caused by drop in temperature dH = –dQ_rev_ = pdV = mgdz = C_p_dT. This simple analytic result for an idealized hypothetical process does not determine the experimental averages of temperature gradients in the atmosphere. 

Likewise, *Newton’s principle of mechanical equilibrium*, i.e., the pressure of an equilibrium fluid in an external field, is the intrinsic pressure plus the weight above with no unbalanced forces. It is central to determining the atmospheric pressure profile that would be given accurately by the barometric pressure (p_B_) but with significant deviations ΔP (z) = p − p_B_ especially in the troposphere, also determined primarily by the variation in temperatures T(z) that can only be a consequence of the radiation balance thermostat.

To complete this preliminary analysis, it is *Gibbs principle of chemical equilibrium* that is the driving force throughout the atmosphere for the direction of all spontaneous changes for all mass transfer events. The Gibbs principle requires uniform chemical potential for every component in the atmosphere everywhere. At pressures of 1 atmosphere and lower, however, the Gibbs energies of nitrogen, oxygen, argon, CO_2_, and other minor gases are essentially the same depending only on the ideal gas concentrations. This is certainly not the case for water and possibly also CO_2_ to a slight extent if there are concentration gradients d[CO_2_]/d_Z_ resulting from its solubility on water.

Mass transfer of water in the atmosphere is the basis of all catastrophic climate change events, floods, and droughts. Whilst one can approximate the driving force for the mass transfer of water in nearly dry unsaturated air by its concentration gradient d[H_2_O]/dz in weather forecast models using Dalton’s law of partial pressures, this is certainly not true for the state changes in atmospheric water between its different states including condensations to its colloidal state in clouds, liquid state in rain, and solid state in snow and ice. All these state changes of water, with latent heat changes, are driven by gradients in T, p, and chemical potential (μ). Non-zero gradients of μ_ι_ is the partial molar Gibbs energy (=dG/dn_i_)_T_ driving force for mass transfer of any single component i of a multicomponent fluid. This essential driving force dμ(H_2_O)/d(z, θ, φ) for extreme weather patterns appears to have been neglected or overlooked as the true driving force in climate change modelling. In a nine-page index to a 453-page treatise on climate change modelling spanning 50 years [8], there is no entry for Gibbs “chemical potential”—the driving force for all spontaneous mass transfer and correlated processes that determine climate and any climate change at the Earth’s lithosphere atmosphere interface.

### 6.2. Thermosphere Measurements

There are two more paradoxical observations in the atmosphere relating to thermodynamic equilibria in the thermosphere. An ever-increasing temperature in the thermosphere and a non-zero wind speed reveals that the outer limit of the thermosphere is apparently rotating faster than the Earth itself. 

At outer limit levels of the thermosphere, however, the rarity of the atmosphere makes measurement of an experimental T(z) by conventional means more difficult. It is necessary to bring the air into thermal equilibrium with the surface of a thermometer that is calibrated to a specific scale [23]. This process takes time. At around an altitude of 100 m, the pressure is so small that the time taken for the gas to equilibrate with the thermometer begins to exceed the observation time for equilibration between gas and thermometer. Recorded temperature apparently higher than the true temperature of thermal equilibrium can be obtained. The ever-increasing T(z) shown in Figure 1 for the thermosphere may be spurious. 

A similar effect apparently occurs in any attempt at this level, ~ 100 km, to measure a long-time average wind velocity which is zero for most of the Earth’s atmosphere where there is no “slip” as the atmosphere rotates with the Earth due to gravity. The equipartition of the energy principle of thermal equilibrium explains both these non-equilibrium effects in the thermosphere. The distribution of molecular velocities in all directions is Maxwellian with a high velocity tail. At extremely low densities when experiments are measured on a finite time scale, the molecular collision frequencies of high velocity molecules are more prominent than low velocity at the thermometer or pressure detecting surface, and fluctuation times diverge. Non-zero ‘average’ wind fluxes from transient finite time measurements become misleading. 

### 6.3. Gravity and Thermal Equilibrium

There appears to be a widespread misapprehension in the modelling literature that a gravitational field *per se* can produce a thermal gradient in an otherwise fluid thermodynamic equilibrium. Classical thermodynamics tells us, however, that the variations in the atmospheric lapse rates cannot be caused by a gravitational potential alone. To confirm this, we appended a formal proof (Appendix A) that a thermal gradient in a fluid in equilibrium in a gravitational field would be a violation of the second law of thermodynamics. Non-zero lapse rates in the atmosphere and temperature gradients were reported in quasi-equilibrium laboratory experiments [24]. As with the more circumspect analysis of atmospheric mean gradients, these laboratory experimental results could be explained by radiation effects.

Changes in the sign of lapse rates are mainly due to variations with z in chemical composition and concentration of the transducer gases. The crossover from a negative to positive lapse rate at the tropopause, for example, is mainly caused by a reduction in the concentration of water above the tropopause and the concentration of ozone of the ozone layer in the stratosphere. 

Finally, we return to the enthalpy ’footprint’ of equation (8). If all the enthalpy from all the fuels (coal, oil, gas, nuclear) burned in the last 100 years emitted into the Earths’ atmosphere at once, what is the temperature rise? The heat capacity C_p_ of the whole atmosphere, using ideal gas value, is 5.6 × 10^21^ J/K; the total ΔH produced by burning fuels from 1920 to 2020 is 3.5 × 10^22^ J. This would increase the global mean <T> by ΔH/C_p_ = 6K (6 degrees centigrade). This is a starting point for the real explanation of global warming!

## Appendix A. Proof That Gravity Cannot Determine Lapse Rates by Reversible Adiabatic Expansion or Any Other Equilibrium Thermodynamic Process

Given the definition of a T-scale [23], and the principles of thermal (T) and mechanical (p) equilibrium:

**[Entropy (S) is a state function: S must remain constant in adiabatic equilibrium process].**
